# 

**Published:** 1885-06

**Authors:** 


OLD SERIES
Vol. XXV.

NEW SERIES
Vol. II.—No. 4.

ESTABLISHED
1855

### The Atlanta Medical and Surgical Journal

Pax et Scientia Sed Veritas Sine timore.

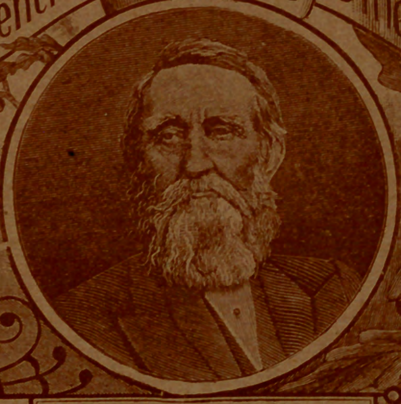

Crawford W. Long M.D.

Edited by W.F. WESTMORELAND. M.D.

H.V.M. MILLER. M.D. LL.D.

JAMES. A. GRAY. M.D.

PUBLISHED BY JAMES. P. HARRISON & CO

ATLANTA, GA.

SUBSCRIPTION: $2.50 A YEAR.

